# Efficacy of thumbtack needle acupuncture combined with PCIA on patients after laparoscopic myomectomy: a randomized controlled trial

**DOI:** 10.3389/fmed.2024.1485211

**Published:** 2024-11-19

**Authors:** Peiyu Mao, Weijie Meng, Tongxin Mao, Hui Li, Xuqun Xu, Xuelu Jiang, Huadi Yang

**Affiliations:** ^1^Department of Gynecology and Obstetrics, The First Affiliated Hospital of Zhejiang Chinese Medical University, Zhejiang Provincial Hospital of Chinese Medicine, Hangzhou, China; ^2^Department of 16# Wards, The First Affiliated Hospital of Zhejiang Chinese Medical University, Zhejiang Provincial Hospital of Chinese Medicine, Hangzhou, China; ^3^First Clinical Medical College, Zhejiang Chinese Medical University, Hangzhou, China

**Keywords:** thumbtack needle acupuncture, PCIA, laparoscopic myomectomy, pain control, postoperative recovery, randomized controlled clinical trial

## Abstract

**Background:**

Pain and gastrointestinal dysfunction after laparoscopic myomectomy (LM) are significant issues that prevent this procedure from being classified as a “Day Surgery.” This study aims to assess the effectiveness and safety of thumbtack needle acupuncture (TNA) combined with patient-controlled intravenous analgesia (PCIA) for promoting enhanced recovery after LM.

**Methods:**

A total of 52 patients were enrolled in the study, divided into a treatment group receiving TNA and a control group. Both groups were administered PCIA without background sufentanil. For rescue analgesia or antiemetic needs, a bolus infusion of flurbiprofen axetil (50 mg) or intravenous metoclopramide (10 mg) was provided. The primary outcomes measured were the Visual Analog Scale (VAS) scores at awakening, as well as at 36 h, 48 h, and 60 h after LM. Secondary outcomes included VAS scores at 6 h, 12 h, 24 h, and 72 h after LM, total sufentanil consumption, the number of PCIA analgesic requests (attempts), the number of doses of rescue flurbiprofen axel analgesia required, preoperative anxiety scores, gastrointestinal function recovery assessment, first ambulation time, length of hospital stay, and patient satisfaction. Adverse events were also recorded.

**Results:**

Compared to the control group, the treatment group showed significantly lower VAS scores, fewer analgesia attempts, reduced total sufentanil consumption, and a smaller number of rescue analgesia doses after LM, along with lower preoperative anxiety scores and higher satisfaction with pain management (*p* < 0.05). Gastrointestinal function recovery was enhanced in the treatment group, as indicated by earlier flatus and defecation, a lower incidence of postoperative nausea and vomiting (PONV), and a smaller number of metoclopramide doses required (*p* < 0.05). Additionally, ambulation occurred earlier, and the length of hospital stay was shorter in the treatment group (*p* < 0.05). No adverse events were observed in patients receiving TNA.

**Conclusion:**

TNA is a safe intervention that effectively alleviates postoperative pain, decreases the total consumption of sufentanil, reduces preoperative anxiety, enhances the recovery of gastrointestinal function, and shortens the duration of hospitalization, making it an ideal adjunct treatment for postoperative recovery after LM. Further research is required to understand the mechanisms underlying this intervention.

**Clinical trial registration:**

www.chictr.org.cn, ChiCTR2300069015.

## Introduction

China, with the largest population in the world, has been facing a shortage of medical resources. Therefore, the concept of enhanced recovery after surgery (ERAS) (1) has gained widespread acceptance among the majority of medical professionals in the country.

Laparoscopic myomectomy (LM) has replaced conventional laparotomy for decades due to its advantages, including less influence on ovarian function, lower oxidative damage, and a higher successful pregnancy rate after operation (2, 3). This procedure is now proficiently performed by many gynecologists and is expected to transition into daytime surgery. However, postoperative pain and gastrointestinal dysfunction prolong hospital stays and escalate medical costs, which prevent it from becoming a “Day Surgery.”

Despite advancements in the prevention and control of postoperative pain over the past 20 years, insufficient analgesia remains a common issue in China. Comprehensive, goal-directed perioperative analgesia (4) is essential to mitigate patients’ traumatic stress responses, especially during often-overlooked phases of preoperative prophylactic analgesia, early postoperative analgesia, and pain management after the withdrawal of patient-controlled intravenous analgesia (PCIA). It is worth noting that preoperative anxiety (5, 6) can also influence the intensity of postoperative pain, anesthesia, and analgesia requirements. Additionally, gastrointestinal dysfunction, including postoperative nausea and vomiting (PONV) and intestinal paralysis caused by anesthesia and analgesics, plays a significant role in affecting postoperative recovery (7, 8).

Thumbtack needle acupuncture (TNA) is a specialized acupuncture therapy that has been used to promote rehabilitation after LM in China. It has attracted clinical attention due to its ease of application, minimal invasiveness, and positive therapeutic effects, although existing evidence supporting its effectiveness remains limited. This study aims to evaluate the effects of TNA during the perioperative period of LM, with the aim of establishing a safe ERAS pathway for this procedure.

## Methods

### Aim

This study aims to assess the efficacy and safety of TNA combined with PCIA without background sufentanil infusion in supporting patient rehabilitation after LM.

### Study design

This prospective, single-center, randomized, participant-blinded, controlled clinical trial was conducted at *The First Affiliated Hospital of Zhejiang Chinese Medical University (Zhejiang Provincial Hospital of Chinese Medicine)* from 1 October 2023, to 30 May 2024, in accordance with the Declaration of Helsinki and the Medical Research Involving Human Subjects Act.

### Setting

All eligible patients scheduled for LM were considered for participation in this study, unless they met the exclusion. The exclusion criteria were as follows: pedunculated subserosal myomas, broad ligament, cervical, or submucosal myomas identified through preoperative ultrasound examination, malignant lesions, use of analgesics within 48 h before surgery, preoperative administration of agents affecting gut motility (e.g., tricyclic antidepressants, opioids, or butylscopolamine), history of preoperative chronic pain, history of gastrointestinal diseases or surgery, previous severe cardiopulmonary disease, liver or kidney dysfunction, history of mental illness, pregnancy or lactation, language and self-expression disorders, limb disability, or smoking habits. The postoperative exclusion criteria were as follows: re-operation exploration, hemodynamic instability, including systolic blood pressure (SBP) <90 mmHg, complications of intervention procedure (fainting when exposed to needles, pain, bleeding, or infection at the needle location), pedunculated subserosal myomas or broad ligament myoma identified through surgery, malignant lesions identified through pathological confirmation, and postoperative complications (severe arrhythmia, hemorrhage, postoperative confusion, urinary tract infection, or reproductive system infection).

### Sample size calculation

In a preliminary trial that evaluated pain scores evaluated using the Visual Analog Scale (VAS) at awakening, as well as at 36 h, 48 h and 60 h, as the main index, it was found that VAS scores at awakening was significantly lower in the treatment group. The treatment group recorded a score of 2.57 ± 0.81, compared to 3.61 ± 1.5 in the control group. This difference achieved the minimal clinically important difference (MCID). Therefore, the sample size calculation was based on VAS scores at awakening. At a two-sided significance level of 0.05 and 90% power, a sample of 104 women was needed (52 per group), accounting for a 15% increase.

### Randomization and masking

Eligible participants were randomly assigned to receive either TNA or control measures using an interactive web response system (IWRS) for clinical research in a 1:1 ratio. Group assignment was conducted exclusively by the acupuncturist (*Nurse Weijie Meng*), who performed the intervention procedure. Clinicians, ward nurses, participants, outcome assessors, and statisticians were all blinded to the treatment allocation.

### Perioperative management and surgery

To minimize bias, we defined standard patient management protocols for all participants in the perioperative periods.

Preoperatively, the patients fasted for 6–8 h before surgery and received an enema in the morning of the surgery.

All LM procedures were performed by our surgical team (consisting of Doctor *Huadi Yang, Peiyu Mao, Xuelu Jiang,* and *Xuqun Xu*), according to standard operating procedures. These surgeries involved the use of a 12-mm, a 10-mm, and two 5-mm laparoscopic trocars and maintenance of intra-abdominal pressure of 11–12 mmHg during the procedure to remove uterine fibroids. A single-shot antibiotic prophylaxis was administered. Sufentanil (0.4 μg/kg), propofol (2–3 mg/kg), and rocuronium (0.6–0.8 mg/kg) were administered intravenously to induce anesthesia. Propofol (3 mg/kg/h), remifentanil (0.1–0.3 μg/kg/min), and sevoflurane (1.0–2.5%) were administered to maintain the depth of anesthesia after tracheal intubation. The bispectral index value was maintained between 40 and 60.

### Intervention procedure

Once the vital signs were stable, the PCIA pumps were connected in the post-anesthesia care unit. Each pump was prepared with 2 μg/kg sufentanil (*Yichang Humanwell Pharmaceutical Jiangxi Province, China*) diluted in 100 mL of physiological saline, set for no background infusion and locked for 6 min. After the setup was complete, the patients were sent back to their wards. The PCIA pumps were discontinued after 36 h for all participants, with sufentanil replenished if depleted within that timeframe. The participants were instructed to use the PCIA pump and were told to press the button (delivering 0.5 mL) whenever they felt pain. For rescue analgesia, a bolus infusion of 50 mg flurbiprofen axel was available if patients reported intolerable pain, which could be repeated as necessary to ensure that analgesia failure was ruled out. Additionally, intravenous metoclopramide 10 mg was administered as a rescue antiemetic upon patients’ request. If severe nausea persisted after two consecutive doses of rescue antiemetics, the PCIA was paused for 2 h and restarted after the symptoms subsided.

**Figure 1:**
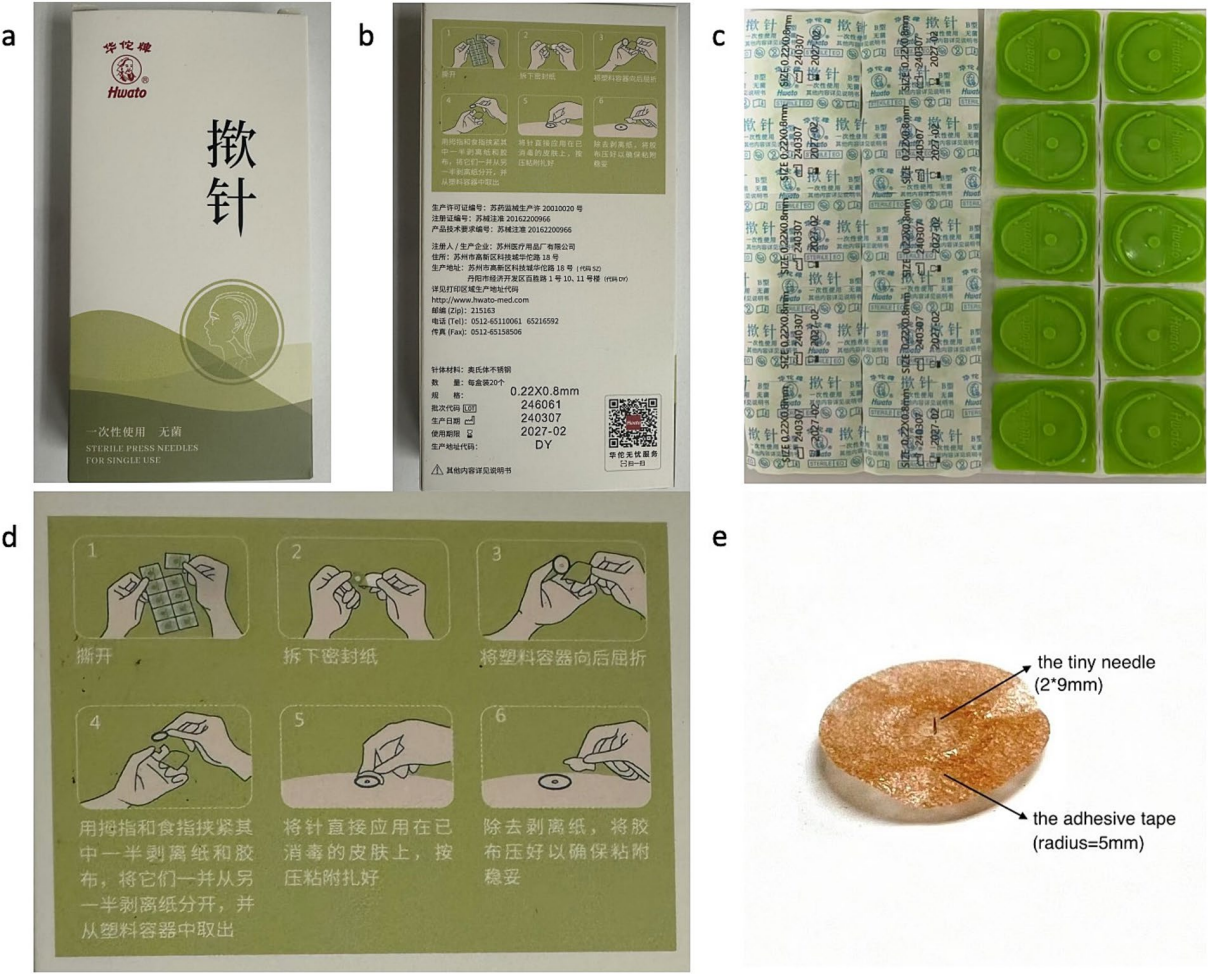
A comprehensive introduction of TNA. (a) Front of packing box of TNA; (b) back of packing box of TNA; (c) packing of TNA in the packing box; (d) the introduction of procedure of TNA on the back of packing box; (e) the TNA consists of a tiny needle and an adhesive tape.

**Figure 2:**
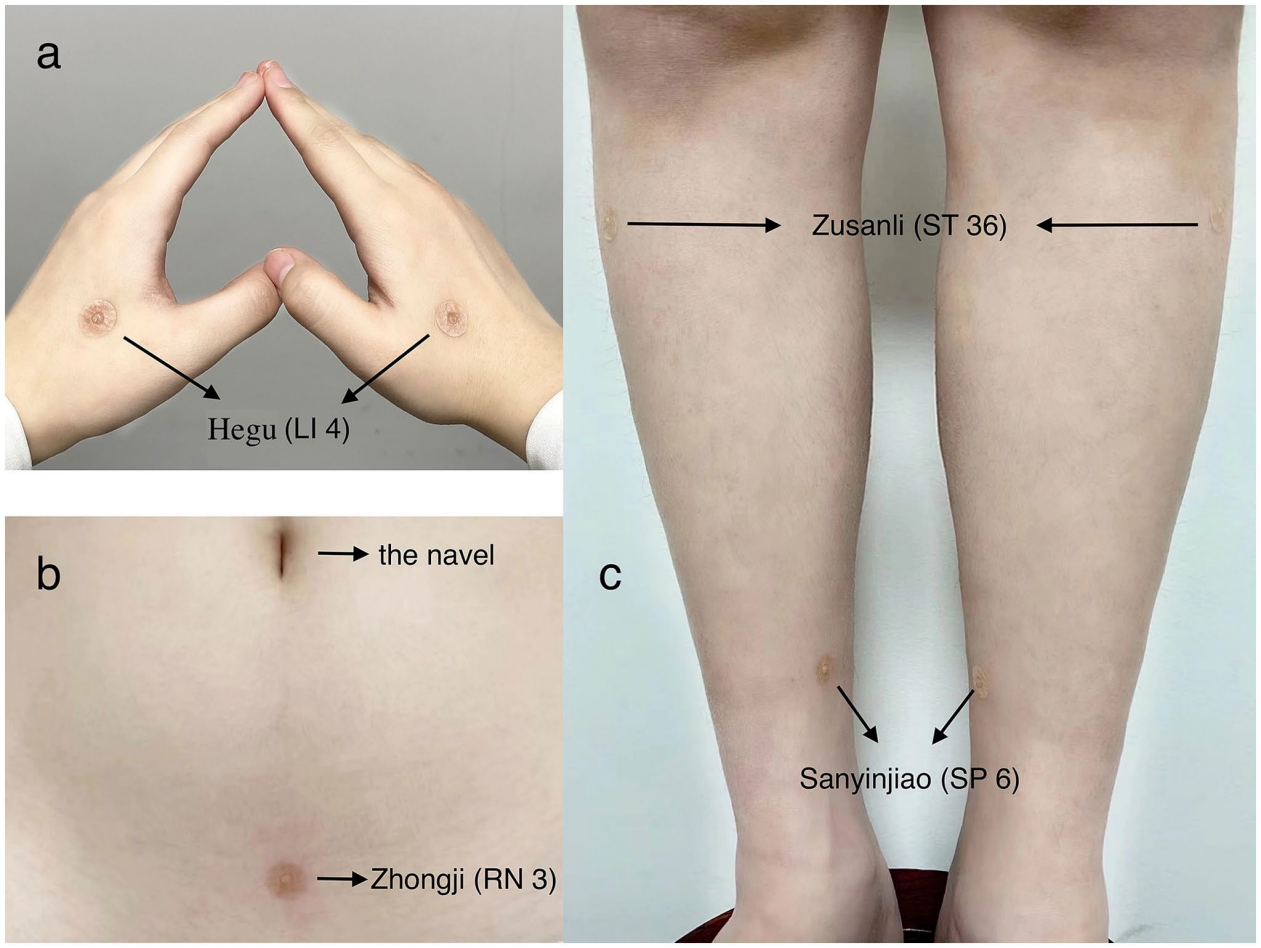
TNA of the marked acupoints. (a) TNA on Hegu (LI 4) of both sides; (b) TNA on the Zhongji (RN 3); (c) TNA on Zusanli (ST 36) and Sanyinjiao (SP 6) of both sides.

TNA (*Huatuo*) used in this study is produced by Suzhou Medical Supplies Factory Co., Ltd., China. Its packing box and the packing of TNA itself are showed in (Figure 1a–c). Figure 1d showed the operation steps through images and Figure 1e showed that TNA consists of a tiny needle (measuring 2 × 9 mm) and a circular adhesive tape with a radius of 5 mm. The night before surgery, *Nurse Meng* performed the intervention for both groups. The acupoints Hegu (LI 4) (9), Sanyinjiao (SP 6), Zusanli (ST 36) (10), and Zhongji (RN 3) were pressed for 10 s, and the superficial skin at these points was disinfected with 75% alcohol. Then, the thumbtack needle was inserted vertically into each acupoint according to the instructions for the treatment group (Figures 1d, 2). In contrast, the control group received only a single acupuncture shot, and an adhesive tape without the tiny needle was placed at the same acupoints for the control group. The intervention procedure was maintained for 48 h. In both groups, *Nurse Meng* reminded the participants to move their limbs normally and to avoid washing or tearing the adhesive tape. To ensure safety, we removed the TNA and immediately reinserted it after the surgery. Postoperative mobilization was standardized in both groups.

Patients with successful defecation, good tolerance to solid meals, no fever in the last 24 h, VAS ≤ 3, full mobilization, no evidence of complications, and who completed the trial were discharged from the hospital.

### Outcome measures

#### Patient characteristics

We collected data on baseline demographics (including marital status, gravidity, parity, previous abdominal operations, age, and body mass index) and surgical characteristics (including the number and size of myomas, pelvic adhesions, operation duration, blood loss, and infusion volume).

#### Primary observation items

VAS scores at awakening, 36 h, 48 h, and 60 h after LM for different types of pain, including visceral pain, incision pain, lower back pain, and shoulder pain, were recorded at awakening, 36 h, 48 h, and 60 h after LM as primary observation items. The intensity of pain was recorded using a 10-point VAS, which ranged from 0 (no pain) to 10 (the worst pain imaginable).

#### Secondary observation items

VAS scores at 6 h, 12 h, 24 h, and 72 h for different types of pain, the total amount of sufentanil consumed, the number of PCIA analgesic requests (attempts), and the number of doses of rescue flurbiprofen axel analgesia required were recorded as secondary pain assessment.

Preoperative anxiety scores assessed using the Hospital Anxiety and Depression Scale (HADS) were recorded.

The first flatus time, the first defecation time, and the incidence of PONV during 48 h after surgery (i.e., nausea refers to an uneasy feeling in the stomach, while vomiting refers to the forceful expulsion of gastric contents (11)), and the number of metoclopramide doses required were recorded to assess gastrointestinal function recovery. PONV is assessed using a four-point verbal scale (none = no nausea, mild = nausea but no vomiting, moderate = vomiting one attack, severe = vomiting > one attack).

First, the ambulation time and the length of hospital stay were recorded as part of the postoperative recovery assessment. Upon discharge, we evaluated patient satisfaction based on four aspects: health education provided by health workers, nursing care, availability of drugs and supplies, and pain management.

#### Adverse events

Adverse events were recorded by *Nurse Meng* and forwarded the information to *Doctor Mao*, who then sent a report to the medical ethics committee to evaluate the case.

### Statistical analyses

Statistical analysis was performed using SPSS® (v. 22.0; IBM Corp., New York, NY) and GraphPad Prism (version 8.0; San Diego, CA, USA) for Windows. Continuous variables are presented as x ®± S. Comparisons between pre-treatment and post-treatment were performed using Student’s *t*-test. Proportions between groups were compared using chi-squared tests, with Fisher’s exact test used as appropriate because of the small sample sizes.

## Results

### Patient characteristics

**Figure 3:**
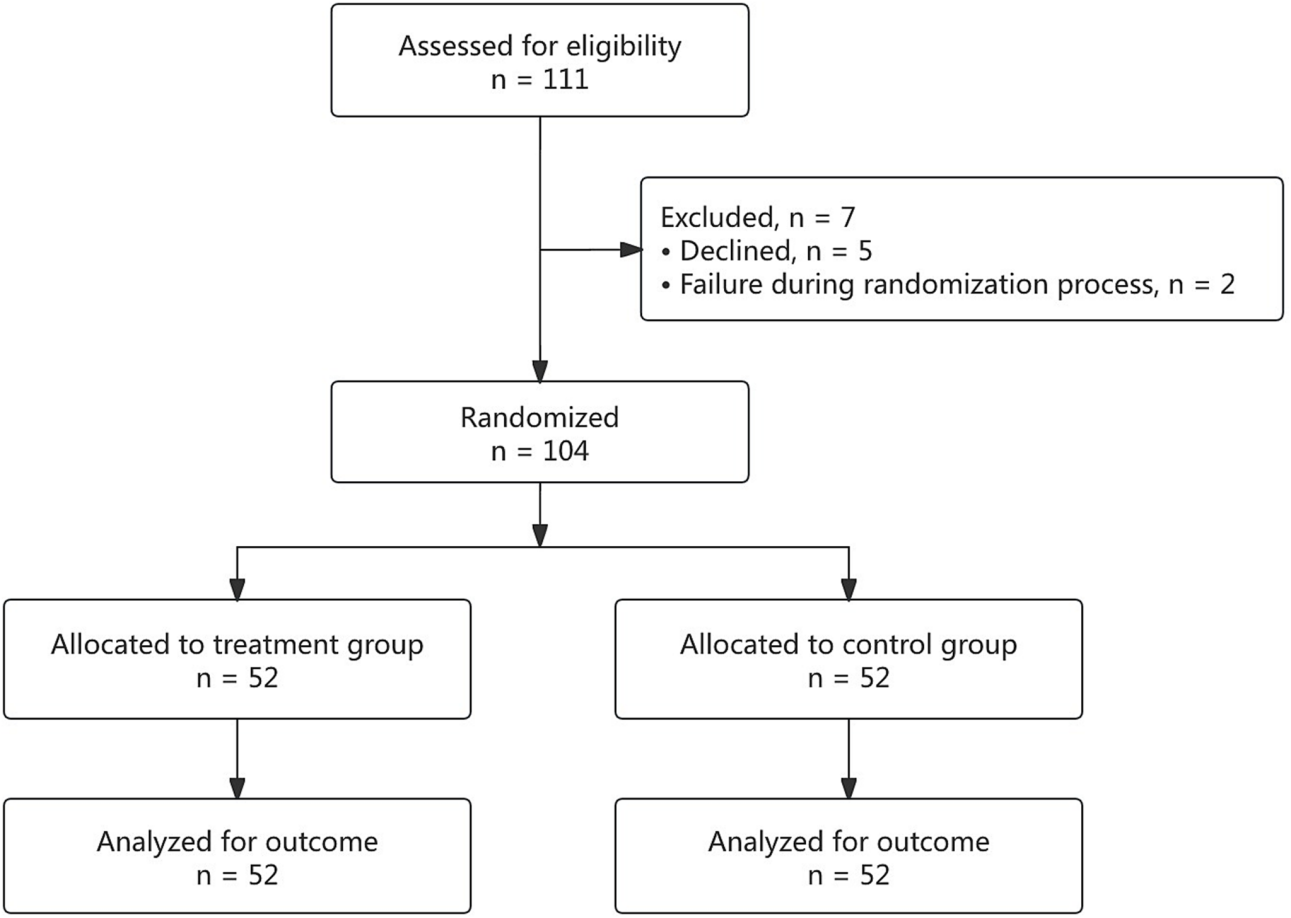
CONSORT flow diagram of the randomized trial.

A total of 111 patients were assessed for eligibility, of which 7 were excluded due to non-compliance with the study protocol. Thus, 104 patients were randomized and completed the trial, with 52 patients in each of the treatment and control groups (Figure 3).

As shown in Table 1, there was no significant difference in baseline demographics and surgical characteristics between the two groups. The postoperative pathology of all participants was confirmed to be benign.

**Table 1:** Preoperative and clinical/surgical characteristics of patients.

Variables	Dimensions	Treatment group	Control group	*p* value
Marital status	Married	48 (92.3%)	50 (96.1%)	0.163^a^
	Single and divorced	4 (7.7%)	2 (3.9%)	
Gravidity	Means ± SD	2.37 ± 1.47	2.98 ± 1.86	0.092^b^
Parity	Means ± SD	1.16 ± 0.64	1.29 ± 0.76	0.327^b^
Previous abdominal operation(s)	0	24 (46.1%)	24 (46.1%)	0.551^c^
	1	25 (48.0%)	22 (42.3%)	
	≥2	3 (5.9%)	6 (11.6%)	
Age	Means ± SD	44 ± 5.90	45 ± 7.43	0.601^b^
Body mass index (kg/m^2^)	Means ± SD	24.60 ± 3.41	23.83 ± 3.67	0.212^b^
Number of myomas removed	1–3	32 (61.5%)	33 (63.5%)	0.883^c^
	4–5	15 (28.9%)	13 (25%)	
	>5	5 (9.6%)	6 (11.5%)	
Diameter of myomas, cm	Means ± SD	7.25 ± 1.72	7.41 ± 1.51	0.626^b^
Pelvic adhesion	YES	29 (55.8%)	28 (53.8%)	>0.999^c^
	NO	23 (44.2%)	24 (46.2%)	
Operation duration, h	Means ± SD	2.33 ± 0.73	2.44 ± 0.92	0.583^b^
Blood loss (range), mL	Means ± SD	53.14 ± 12.57	53.14 ± 12.57	0.940^b^
Infusion volume intraoperatively (range), mL	Means ± SD	1064.71 ± 154.69	1064.71 ± 154.69	>0.999^b^

^aFisher’s exact test, bStudent’s t-test, cChi-Squared test.^

### Primary outcome measures

Table 2 shows that compared with the control group, the treatment group had significantly lower VAS scores for incision and shoulder pain at awakening, shoulder pain at 36 h, and all pain types at 48 h and 60 h (*p* < 0.05). In both groups, incision pain was the highest at awakening, visceral pain was the highest at 36 h and 48 h, and shoulder pain was the highest at 60 h.

**Table 2:** VAS scores at awakening time, 36 h, 48 h, and 60 h after surgery for two groups.

Time	Pain types	VAS scores		*P* value
		Treatment group (*n* = 52)	Control group (*n* = 52)	
Awakening time	Visceral pain	2.81 ± 0.56	2.94 ± 0.64	0.257^a^
	Incision pain	3.88 ± 0.78	4.21 ± 0.72	0.029^a^
	Lower back pain	2.69 ± 0.54	2.88 ± 0.47	0.057^a^
	Shoulder pain	2.77 ± 0.58	3.00 ± 0.56	0.042^a^
36 h	Visceral pain	2.60 ± 0.98	2.90 ± 0.80	0.081^**a**^
	Incision pain	2.56 ± 0.83	2.85 ± 0.78	0.070^**a**^
	Lower back pain	2.50 ± 0.73	2.71 ± 0.64	0.118^**a**^
	Shoulder pain	2.37 ± 0.60	2.62 ± 0.57	0.030^**a**^
48 h	Visceral pain	2.54 ± 1.18	2.98 ± 0.90	0.034^a^
	Incision pain	2.29 ± 2.00	2.62 ± 0.63	0.020^a^
	Lower back pain	2.27 ± 0.60	2.56 ± 0.75	0.033^a^
	Shoulder pain	2.27 ± 0.93	2.62 ± 0.69	0.034^a^
60 h	Visceral pain	1.83 ± 0.68	2.15 ± 0.70	0.017^a^
	Incision pain	1.75 ± 0.62	2.02 ± 0.64	0.032^a^
	Lower back pain	1.67 ± 0.58	1.94 ± 0.61	0.010^a^
	Shoulder pain	2.04 ± 0.82	2.35 ± 0.62	0.033^a^

^aStudent’s t-test. Values are given as means ± SD.^

### Secondary pain assessment

As shown in Tables 3, 4, the treatment group exhibited significantly lower VAS scores for shoulder pain at 6 h, 12 h, and 24 h, as well as for all pain types at 72 h, compared to the control group. Additionally, the treatment group had fewer attempts, reduced total sufentanil consumption, and a fewer doses of rescue analgesia (*p* < 0.05).

**Table 3:** VAS scores at 6 h, 12 h, 24 h, and 72 h after surgery for two groups.

Time	Pain types	VAS scores		*P* value
		Treatment group (*n* = 52)	Control group (*n* = 52)	
6 h	Visceral pain	2.75 ± 0.76	3.00 ± 0.66	0.077^a^
	Incision pain	2.46 ± 0.61	2.65 ± 0.59	0.105^a^
	Lower back pain	2.67 ± 0.68	2.87 ± 0.56	0.118^a^
	Shoulder pain	2.56 ± 0.64	2.87 ± 0.69	0.020^a^
12 h	Visceral pain	2.38 ± 0.69	2.62 ± 0.66	0.085^a^
	Incision pain	2.35 ± 0.68	2.58 ± 0.67	0.084^a^
	Lower back pain	2.17 ± 0.76	2.38 ± 0.69	0.140^a^
	Shoulder pain	2.12 ± 0.68	2.38 ± 0.63	0.038^a^
24 h	Visceral pain	2.23 ± 0.73	2.48 ± 0.61	0.061^a^
	Incision pain	2.13 ± 0.71	2.35 ± 0.59	0.103^a^
	Lower back pain	2.00 ± 0.69	2.23 ± 0.61	0.074^a^
	Shoulder pain	2.52 ± 0.78	2.88 ± 0.88	0.027^a^
72 h	Visceral pain	1.65 ± 0.68	1.98 ± 0.75	0.022^a^
	Incision pain	1.77 ± 0.73	2.10 ± 0.87	0.040^a^
	Lower back pain	1.56 ± 0.61	1.83 ± 0.68	0.035^a^
	Shoulder pain	1.48 ± 0.58	1.75 ± 0.65	0.028^a^

^aStudent’s t-test. Values are given as means ± SD.^

**Table 4:** Attempts, total sufentanil consumption, and number of rescue analgesia/antemetic, preoperative anxiety, time of flatus, defecation and ambulation, and hospital stay for two groups.

Variable		Treatment group (*n* = 52)	Control group (*n* = 52)	*P* value
Attempts		13.85 ± 4.43	16.27 ± 5.41	0.014^a^
Total sufentanil consumption (μg.)		0.12 ± 0.04	0.15 ± 0.07	0.013^a^
Number of rescue analgesia	1	4 (6.7%)	1 (9.1%)	0.035^b^
	2	2 (33.3%)	7 (63.6%)	
	≥3	0 (0.0%)	3 (27.3%)	
HADS scores of anxiety		3.40 ± 1.0	3.79 ± 0.80	0.032^a^
Time to first flatus (h)		13.67 ± 3.92	15.18 ± 3.06	0.022^a^
Time to first defecation (h)		23.08 ± 3.75	24.86 ± 3.24	0.014^a^
The incidence of PONV		6 (11.5%)	15 (28.8%)	0.049^a^
Number of metoclopramides required	1	4 (80.0%)	2 (6.7%)	0.044^b^
	2	1 (20.0%)	9 (75%)	
	≥3	0 (0.0%)	1 (8.3%)	
Time to ambulation (h)		15.76 ± 2.29	17.27 ± 3.62	0.014^a^
Length of hospital stay (d) after LM		4.00 ± 0.89	5.00 ± 0.99	0.030^a^

^a^Student’s *t*-test. Continuous data are shown as mean ± SD, enumeration data are shown as number (percentage).

### Preoperative anxiety assessment

As shown in Table 4, the HADS scores for anxiety were significantly lower in the treatment group (*p* < 0.05).

### Gastrointestinal function recovery assessment

As shown in Table 4, gastrointestinal function recovery was enhanced in the treatment group with earlier flatus and defecation (*p* < 0.05), a lower incidence of PONV, and a fewer doses of metoclopramide required (*p* < 0.05).

### First ambulation time and hospital stay assessment

As shown in Table 4, the time of ambulation was earlier and the length of hospital stay was shorter in the treatment group (*p* < 0.05).

### Patient satisfaction assessment

As shown in Table 5, there was no significant difference in satisfaction with the health education provided by health workers, nursing care, and availability of drugs and supplies. However, the satisfaction score for pain management in the treatment group was significantly higher than that in the control group (*p* < 0.01).

**Table 5:** Participants’ satisfaction outcomes.

Variables	Groups	Very satisfied (5)	Satisfied (4)	Neutral (3)	Dissatisfied (2)	Very dissatisfied (1)	Means ± SD	*P* value
Health education provided by the health workers	Treatment group	3 (5.8%)	36 (69.2%)	10 (19.2%)	2 (3.8%)	1 (2.0%)	3.73 ± 0.72	0.826^a^
	Control group	3 (5.8%)	36 (69.2%)	10 (19.2%)	2 (3.8%)	1 (2.0%)	3.65 ± 0.79	
Nursing care	Treatment group	4 (7.7%)	35 (67.3%)	12 (23.1%)	1 (1.9%)	0 (0.0%)	3.78 ± 0.58	>0.999^a^
	Control group	3 (5.7%)	37 (71.2%)	11 (21.2%)	1 (1.9%)	0 (0.0%)	3.80 ± 0.57	
Availability of drug and supply	Treatment group	6 (11.5%)	34 (65.4%)	11 (21.2%)	1 (1.9%)	0 (0.0%)	3.86 ± 0.63	0.515^a^
	Control group	3 (5.7%)	36 (69.2%)	12 (23.1%)	1 (2.0%)	0 (0.0%)	3.78 ± 0.58	
Pain management	Treatment group	10 (19.2%)	20 (38.5%)	21 (40.4%)	1 (1.9%)	0 (0.0%)	3.75 ± 0.80	<0.0001^a^
	Control group	2 (3.8%)	10 (19.2%)	35 (67.3%)	3 (5.7%)	2 (4.0%)	3.14 ± 0.75	

^aStudent’s t-test.^

### Adverse events

No significant intraoperative or postoperative complications were observed in both groups. TNA therapy was well tolerated. No complications such as skin allergies, fainting when exposed to needles, pain, bleeding, or infection at the needle location were noted.

## Discussion

This study aims to investigate the benefits and safety of TNA combined with PCIA for patients after LM. According to the American Pain Society postoperative guidelines (12), the use of background infusion is not required due to the potential aggravation of PONV. Therefore, PCIA was administered without a background infusion of sufentanil in this trial.

Although PCIA pumps are commonly used to manage postoperative pain, pain during the awakening period and after PCIA discontinuation is frequently overlooked due to healthcare providers’ busy schedules. Even if a ward nurse or surgeon provides rescue analgesic treatment at the patient’s request, the effect is often unsatisfactory, followed by constant complaints. Inadequate pain management has emerged as a primary cause of dissatisfaction among postoperative patients and their families. It is also an important cause of postoperative acute pain evolving into chronic pain and an important blind spot that has not yet been taken seriously by surgeons and anesthesiologists. Besides, although opioids can control incision and visceral pain, they cannot effectively control post-laparoscopic shoulder pain (13). A previous study (14) reported that TNA combined with PCIA obtained better analgesic effects on incision dynamic pain and uterine contraction pain in patients after cesarean section. In this trial, we confirmed that TNA not only significantly alleviates postoperative incision and visceral pain but also alleviates shoulder pain. The application of TNA can cover the analgesic blind spots: preoperative prophylactic analgesia, early awakening analgesia, and pain after withdrawal of PCIA. Milder pain levels result in lower sufentanil use, reducing opioid-related adverse events (15), especially respiratory depression and postoperative ileus.

Preoperative anxiety is another factor that influences the intensity of postoperative pain and analgesia requirements. Our findings indicate that patients receiving TNA reported lower HADS anxiety scores. It is reported (6) that patients with preoperative anxiety could benefit from multimodal analgesia, including non-pharmacological methods. Therefore, relieving anxiety may be another analgesic mechanism of TNA.

PONV and constipation are classical side effects of opioids (7, 16), which are other difficulties that need to be solved in the ERAS context. With the return of patients’ water intake and upcoming PONV pathophysiological climax (24 h postoperatively), PONV occurs more frequently and intensively (17). In this trial, we also found that the first flatus time and first defecation time were shorter, and the incidence of PONV during postoperative 48 h was lower in patients with TNA, showing enhanced recovery of gastrointestinal function, which is related to the less consumption of sufentanil and may relate to the mechanism of TNA itself. A pragmatic randomized controlled trial study (18) of TNA on functional constipation has shown that compared with mosapride citrate, TNA produced a greater improvement in complete and spontaneous bowel movement scores, consistent with this study’s results.

Earlier ambulation was also observed in the treatment group, which is related to better pain control. It is reported (19) that early ambulation can reduce stress response and pain and accelerate patients’ recovery, creating a good mutually reinforcing cycle.

Notably, the outcomes of TNA therapy largely depend on the patient’s cooperation. In this study, the patients showed good tolerance to the treatment. The TNA procedure was brief, caused minimal discomfort, and showed no adverse reactions, demonstrating its safety. In addition, as a non-drug treatment, it is widely accepted by patients with liver and kidney dysfunction. Reduced pain and faster recovery led to better pain control, shorter hospital stays, and lower medical costs.

Traditional Chinese medicine views postoperative pain (20) as a result of “meridian obstruction, which can alleviated by clearing the meridian pathways (21). Stimulating acupoints serves as a method to facilitate this process, regulating the cerebral cortex and calming the nerves, thereby reducing swelling and alleviating pain (22).

TNA maintains continuous acupoint stimulation, promoting the flow of “Qi,” a Chinese concept representing vitality or energy while relieving tension and alleviating associated symptoms (23). Several trials have demonstrated the effectiveness of TNA in various contexts, including pain management during obstetric delivery (24), chronic pain conditions (25), nausea and vomiting (26), and dysmenorrhoea (27). Our study confirmed its efficacy in managing pain and enhancing gastrointestinal function recovery after LM.

Multimodal analgesia is currently highly valued in the ERAS era. However, its implementation can be challenging for surgeons and anesthesiologists in China due to the demands of a busy medical environment. Therefore, TNA, performed by nurses, can serve as an integral component of multimodal analgesia to enhance recovery after LM.

## Conclusion

TNA is a safe intervention that effectively alleviates postoperative pain, reduces the total consumption of sufentanil, relieves preoperative anxiety, improves the recovery of gastrointestinal function, and shortens hospitalization duration, making it an ideal adjunct treatment for the postoperative recovery of LM. Further research is required to understand the mechanisms underlying this intervention.

## Data Availability

The original contributions presented in the study are included in the article/supplementary material. Further inquiries can be directed to the corresponding author.
